# Cardiovascular surgery experience does not significantly improve patients' response to stroke

**DOI:** 10.1002/brb3.1405

**Published:** 2019-09-12

**Authors:** Shengde Li, Li‐Ying Cui, Craig Anderson, Chunpeng Gao, Chengdong Yu, Guangliang Shan, Longde Wang, Bin Peng, the FAST‐RIGHT Investigators and Coordinators, Nan Jiang, Yuehui Hong, Weidong Liu, Jian Li, Suiqiang Zhu, Ping Xu, Tiemin Wei, Yun Luo, Shengli Chen, Dan Liu, Dongmei Xie, Dong Xu, Fei Wei, Guanghui Wu, Hongyan Li, Hua Luo, Jie Min, Jinhai Tang, Jun Sun, Luoqing Li, Qi Yao, Shilin Liu, Wei Shi, Wei Yan, Xiaofei Yu, Xiaopeng Luo, Xiaoxiang Peng, Ya Zhang, Yang Gao, Ye Peng, Yongling Xue, Zhi Lin

**Affiliations:** ^1^ Department of Neurology Peking Union Medical College Hospital Peking Union Medical College and Chinese Academy of Medical Sciences Beijing China; ^2^ Neurological and Mental Health Division The George Institute for Global Health Faculty of Medicine University of New South Wales Sydney Australia; ^3^ The George Institute for Global Health Peking University Health Science Center Beijing China; ^4^ Disease Control and Prevention Office Dalian Municipal Central Hospital Liaoning China; ^5^ Department of Epidemiology and Statistics Institute of Basic Medical Sciences Chinese Academy of Medical Sciences Beijing China; ^6^ Stroke Control Project Committee The National Health Commission Beijing China

**Keywords:** awareness, cardiovascular surgical procedures, emergency medical services, health education, patient compliance, stroke

## Abstract

**Objectives:**

Patients with a history of cardiovascular surgery are at risk of stroke, and immediately calling emergency medical services (EMS) after stroke onset is crucial to receiving effective reperfusion therapy. We aimed to determine the effect of a history of cardiovascular surgery on patients' ability to recognize stroke and intent to call EMS.

**Methods:**

We performed a cross‐sectional community‐based study from January 2017 to May 2017. A total population of 186,167 individuals, recruited from 69 administrative areas across China, was analyzed. Different multivariable logistic regression models were performed to identify the associations between cardiovascular surgical history and stroke recognition or intent to call EMS, respectively.

**Results:**

0.1% of the total population had a history of cardiovascular surgery. In the surgery group, the estimated stroke recognition rate (SRR) and correct action rate (CAR) were 84.9% and 74.7%, respectively. The prevalence of cardiovascular risk factors was significantly higher in the surgery group. Cardiovascular surgical history was not associated with recognition of stroke across different models. The surgery group was more likely to call EMS, but the difference was not significant after full adjustment (OR: 1.40, 95% CI: 0.99–1.98, *p* = .0572).

**Conclusions:**

Cardiovascular surgical history does not influence patients' likelihood of calling EMS more often at stroke onset. Patients receiving cardiovascular surgeries should be counseled regarding stroke recognition, proper response to stroke, and the importance of controlling risk factors.

## Graphical abstract

Only 0.1% of the total population had a history of cardiovascular surgery. However, cardiovascular surgical history does not influence patients' likelihood of calling EMS more often at stroke onset.

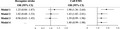

## 1 INTRODUCTION

Carotid artery stenosis, coronary artery disease, and cerebral hemorrhage are associated with incident stroke (Kim et al., 2017; Mathiesen, Joakimsen, & Bonaa, 2001; Nielsen, Larsen, Skjoth, & Lip, 2017; Sobiczewski, Wirtwein, Trybala, & Gruchala, 2013). Many cardiovascular interventions, such as carotid endarterectomy, carotid artery stenting, extracranial–intracranial bypass, coronary artery bypass grafting, and percutaneous transluminal coronary intervention, were introduced to treat artery stenosis (Anderson & Morrow, 2017; Kernan et al., 2014). However, even after these interventions, patients still have a markedly higher stroke risk (Hobeanu et al., 2017), and their compliance does not improve (Halle, Benarroch‐Gampel, Teodorescu, & Rajani, 2018). Early presentation to medical care is crucial to receiving effective thrombolysis, which improves outcomes from acute ischemic stroke (Emberson et al., 2014; Saver et al., 2010). Calling emergency medical services and direct ambulance transfer would promote access to thrombolysis (Fonarow et al., 2011; Jin et al., 2012). It is commonly assumed that patients with cardiovascular surgery history know about stroke risk and how to respond to stroke symptoms. However, the impact of cardiovascular interventions on stroke knowledge remains unknown. The aim of this study was to examine the effect of cardiovascular surgery on stroke recognition and calling EMS.

## 2 METHODS

### 2.1 Study design and participants

This study was based on the FAST‐RIGHT study, a part of the China National Stroke Screening Survey (CNSSS), which had been described elsewhere (Li et al., 2019; Longde et al., 2015; National Center for Stroke Control & Prevention, the National Health Commission, 2016). In brief, the CNSSS was a national cross‐sectional community‐based survey with a 2‐stage stratified sampling framework, screening adults aged 40 years and older in each community in 221 administrative areas in 31 provinces of Mainland China. The FAST‐RIGHT study enrolled a subgroup of residents from 69 administrative areas and conducted face‐to‐face interviews between January 2017 and May 2017 (Li et al., 2019). In addition to standard questionnaires, participants were asked four questions about their awareness of strokes and responses pertaining to them (details are outlined in the Appendix S1(2); Harbison et al., 2003). The FAST‐RIGHT study was approved by the central ethics committee of Peking Union Medical College Hospital (the principal study center), and written informed consent was obtained from all participants.

### 2.2 Explanatory and outcome variables

The assessments were conducted by trained staff with a standard questionnaire, covering socio‐demographic, medical, family history, and lifestyle factors. Additionally, there was a physical examination which included cardiac auscultation and measurements of height, weight (for calculation of body mass index [BMI]), and waist circumference. In the FAST‐RIGHT study, four questions about stroke awareness were asked, and additional professional training was provided to project‐related staff. After completion of the questionnaire survey, stroke education was provided. All screening data were transferred from questionnaires to an electronic database and checked centrally for completeness and errors by an experienced data manager.

The participant's unprovoked awareness of “facial droop,” “arm weakness,” and “speech disturbance (slurred speech or word‐finding difficulties)” was regarded as recognition of stroke symptoms (Harbison et al., 2003). Correct action in response to stroke was defined as calling EMS immediately after the onset of any of these symptoms. The risk factors assessed in the questionnaire included hypertension, diabetes mellitus, dyslipidemia, atrial fibrillation (AF)/valvular heart disease, overweight/obesity, smoking, physical inactivity, and family history of stroke (Appendix S1(3)), based on the standard definitions (Longde et al., 2015). Stroke history was established by self‐reporting and confirmed by a neurologist or physician using neuroimaging, according to standard diagnostic criteria. Participants were stratified into five stroke risk categories: stroke, previous transient ischemic attack (TIA), and “high,” “moderate,” and “low” risk (Appendix S1(4)).

Carotid endarterectomy, carotid artery stenting, extracranial–intracranial bypass, coronary artery bypass grafting, percutaneous transluminal coronary intervention, and intracranial hemorrhage surgery were classified as cardiovascular surgery. The total population was divided into two subgroups: participants with a history of any of the above surgeries were placed in the surgery group, while those without any cardiovascular surgical history were assigned to the nonsurgery group.

### 2.3 Statistical analysis

The socio‐demographic data of the surgery and nonsurgery groups were presented as absolute values with proportions. Pearson's chi‐square tests were performed to compare socio‐demographic data between the surgery and nonsurgery groups. Overall stroke recognition rate (SRR) and correct action rate (CAR) were presented as rates in the surgery and nonsurgery groups, separately. The associations between history of cardiovascular surgery and recognition of stroke and calling EMS were analyzed using multivariable logistic regression. Different models were established to adjust for other potential confounders. All potential confounding variables considered for regression were classified into the following six categories: Demographic data (including age, sex, site, and region), Social status (including annual income and education level), Family‐related conditions (including number of children, living situation, and the patient's experience (if any) with stroke in relatives or colleagues), Cardiovascular risk factors (including hypertension, diabetes mellitus, dyslipidemia, atrial fibrillation (AF)/valvular heart disease, overweight/obesity, smoking, physical inactivity, family history of stroke, and previous cerebral vascular disease), Number of avenues to learn about stroke, and Recognition of stroke symptoms. All analyses were performed using SAS version 9.3, and a standard 2‐sided *p* value (<.05) was considered statistically significant.

## 3 RESULTS

**Figure 1:**
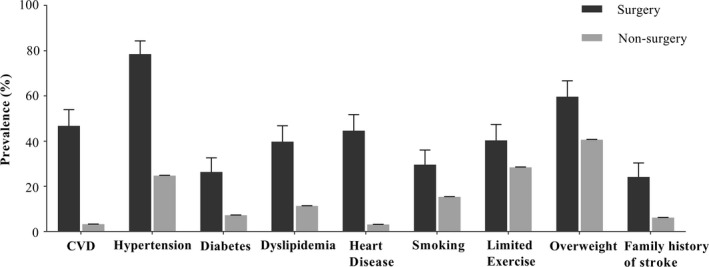
Prevalence of cardiovascular risk factors between cardiovascular surgery and nonsurgery groups. CVD: Adults with history of cerebral vascular disease, including ischemic stroke, cerebral hemorrhage, subarachnoid hemorrhage, and transient ischemic attacks. Overweight indicates body mass index (BMI):24–50

Of 187,723 participants, 1556 were excluded due to lack of information on surgical history (Figure S1 and Table S1 in Appendix S1). Among the remaining 186,167 residents, 186 (0.1%) received cardiovascular surgery, while 185,981 (99.9%) did not (nonsurgery group). Age, sex, urban–rural site, region, annual income, and the patient's experience (if any) with stroke in relatives or colleagues differed significantly between the surgery and nonsurgery groups (Table 1). The overall rates of stroke recognition rate and correct action rate in the surgery group were 84.9% and 74.7%, respectively (Table 2). The main reason for not calling an ambulance was waiting for a family member (23.7% in surgery group vs. 36.0% in nonsurgery group). Table 1 also shows higher proportions of elderly, male, and urban residents in the surgery group. The prevalence of stroke in the surgery group was 46.8%, 14.3 times that in residents without history of cardiovascular surgery (3.3%; Figure 1). Similarly, cardiovascular risk factors in the surgery group were significantly more prevalent at about 1.4–14.3 times those in the nonsurgery group (Figure 1). The proportion of high‐risk level and above for stroke in the surgery group was triple that of the nonsurgery population (76% vs. 25%; Figure S2 in Appendix S1).

**Table 1:** Socio‐demographics characteristics of the surgery and nonsurgery groups

	Surgery, *n* (%)	Nonsurgery, *n* (%)	*p* Value
Age, years			
40–49	10 (5.4)	38,862 (20.9)	<.0001
50–59	32 (17.2)	50,570 (27.2)	
60–69	69 (37.1)	51,381 (27.6)	
70–79	58 (31.2)	31,931 (17.2)	
80–99	17 (9.1)	13,196 (7.1)	
Sex			
Male	114 (61.3)	84,419 (45.4)	<.0001
Female	72 (38.7)	101,562 (54.6)	
Site			
Urban	115 (61.8)	89,868 (48.3)	.0002
Rural	71 (38.2)	96,113 (51.7)	
Regions			
North + Northeast	26 (14.0)	10,864 (5.8)	<.0001
East	35 (18.8)	55,379 (29.8)	
Central	77 (41.4)	59,705 (32.1)	
South	28 (15.0)	22,488 (12.1)	
Southwest	15 (8.1)	20,574 (11.1)	
Northwest	5 (2.7)	16,971 (9.1)	
Education			
≤Primary	69 (37.1)	85,577 (46.0)	.0504
Middle/High school	105 (56.4)	89,679 (48.2)	
≥College	12 (6.5)	10,720 (5.8)	
Personal Annual Income (US $)			
<731	51 (27.6)	51,962 (27.9)	.0184
731–2,923	51 (27.6)	67,301 (36.2)	
>2,923	83 (44.8)	66,669 (35.9)	
Living status^a^			
With family	180 (97.3)	179,094 (96.3)	.4812
With others	5 (2.7)	6,838 (3.7)	
Children number			
0	3 (1.6)	1,540 (0.8)	.1244
1	44 (23.8)	48,259 (26.0)	
2–3	106 (57.3)	112,972 (60.8)	
≥4	32 (17.3)	22,972 (12.4)	
Stroke in others^b^			
No	104 (55.9)	154,397 (83.0)	<.0001
Yes	82 (44.1)	31,582 (17.0)	
Avenues^c^			
1	91 (48.9)	93,457 (50.3)	.5495
2–3	83 (44.6)	83,653 (45.0)	
4–6	12 (6.5)	8,840 (4.7)	

a With family includes living with spouse/children; with others include being single, living in a nursing home, and with other people.

b Relatives or colleagues who have suffered an acute stroke.

c Number of avenues taken to learn about acute stroke.

**Table 2:** Stroke recognition rate (SRR) and correct action rate (CAR) in the surgery and nonsurgery groups

	SRR *n*/*N* (%)	*p* Value	CAR *n*/*N* (%)	*p* Value
Surgery				
No	152,040/185,981 (81.8,81.6–81.9)	.2593	112,962/185,981 (60.7, 60.5–61.0)	<.0001
Yes	158/186 (84.9, 79.8–90.1)		139/186 (74.7,68.5–81.0)	

Note

*N*, total in every variable; *n*, number for recognizing stroke/correct action to stroke.

**Figure 2:**
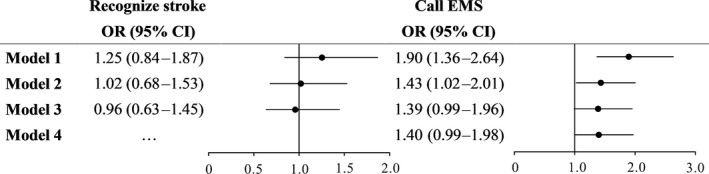
Different logistic regression models presenting the associations between cardiovascular surgery and stroke recognition or calling EMS. EMS, emergency medical service. Model 1: Unadjusted. Model 2: Adjusted by demographic data and cerebral vascular disease, hypertension, diabetes, and dyslipidemia. Model 3: Adjusted by demographic data, cerebral vascular disease, hypertension, diabetes, and dyslipidemia, social status, living status, and number of avenues to learn about stroke. Model 4: Adjusted by demographic data, cerebral vascular disease, hypertension, diabetes, and dyslipidemia, social status, living status, number of avenues to learn about stroke, and stroke recognition for calling EMS

Univariate analysis shows that only the rate of calling EMS was significantly higher in the surgery group than in the nonsurgery population. The surgery group tended to call EMS at the time of stroke symptoms in the multivariate analysis, but this association was not significant statistically after full adjustment (OR: 1.40, 95% CI: 0.99–1.98, *p* = .0572; Figure 2). In addition, a history of cardiovascular surgery did not increase the odds of recognizing stroke symptoms consistently across 3 different logistic models.

In subgroup analysis of surgery group, the patients with cerebra vascular disease seemed more likely to recognize stroke (86.2% vs. 83.8%) and call EMS (77.0% vs. 72.7%), but without statistical significance (Table 3). Compared with rural group, urban individuals showed markedly higher intent to call EMS in both nonsurgery (70.2% vs. 51.9%) and surgery (80.0% vs. 66.2%) groups (Table S2 in Appendix S1).

**Table 3:** Stroke recognition rate (SRR) and correct action rate (CAR) in individuals with and without CVD among the surgery group

	SRR *n*/*N* (%)	*p* Value	CAR *n*/*N* (%)	*p* Value
CVD				
No	83/99 (83.8, 76.6–91.1)	.6522	72/99 (72.7, 64.0–81.5)	.5023
Yes	75/87 (86.2, 79.0–93.5)		67/87 (77.0, 68.2–85.9)	

Note

*N*, total in every variable; *n*, number for recognizing stroke/correct action to stroke.

Abbreviation: CVD, cerebral vascular disease.

## 4 DISCUSSION

Our study is the first to present that the surgery interventions did not improve patients' awareness of stroke or prompt them to respond to stroke onset properly. Nearly three‐fourths of patients stated that they would call an ambulance for stroke onset in the questionnaire survey. However, we should note that in clinical practice in China, only 21.5%–25% of stroke patients arrived at hospital within 3 hr, and the overall rate of using an ambulance was 15.4%–23.1% (Jiang et al., 2016; Jin et al., 2012; Wang et al., 2011; Yin et al., 2016). In a real scenario, individuals may be nervous, disturbed, or misled by others who deny or minimize stroke symptoms (Teuschl & Brainin, 2010). As cardiovascular surgery did not significantly increase the odds of calling an ambulance (Fonarow et al., 2011), we do not think that the surgery group will perform better at calling an ambulance than the nonsurgery group.

Contrary to commonly held belief, substantial risk of arterial disease complications still exists after carotid intervention (Hobeanu et al., 2017), wherein the majority of strokes occur without significant restenosis or occlusion (Kumar et al., 2017). In addition, modifiable risk factors like hypertension, diabetes, abnormal lipids, limited physical activity, obesity, smoking, alcohol consumption, cardiac disease, unhealthy diet, and psychosocial factors account for most strokes (O'Donnell et al., 2016), and are also risk factors for myocardial infarction (Yusuf et al., 2004). Unfortunately, even cardiac patients underestimated the role of cardiovascular risk factors (Soroush et al., 2017). In fact, even if receiving cardiovascular intervention, patients were still at a high risk for stroke due to their markedly high risk factors and poor control of hypertension, diabetes, and dyslipidemia (Lu et al., 2017; Pan et al., 2016; Qin et al., 2016).

It was our limitation that we did not distinguish surgery procedure for stroke or nonstroke reasons. However, some of the individuals received cardiovascular surgery interventions due to nonstroke disorders; these procedures were performed in departments of intervention, vascular surgery, neurosurgery, cardiology, and cardiac surgery. We speculate that it is hard for them to think of stroke onset in the future, though in fact carotid artery stenosis and coronary heart disease both increase the stroke risk and indicate the presence of multiple risk factors (Anderson & Morrow, 2017; Mathiesen et al., 2001; Sobiczewski et al., 2013; Yusuf et al., 2004). Actually, less than half of the patient population understood that carotid endarterectomy was aimed to prevent future stroke (Maruthappu et al., 2010). Only 31.5% of patients would discuss their long‐term stroke risk with surgeons when they decided to undergo carotid endarterectomy, markedly lower than those rejecting surgery (72.4%; Middleton, Gattellari, Harris, & Ward, 2006). Although more than four‐fifths of the patients in the surgery group were able to identify symptoms of stroke onset, they did not respond better than the nonsurgery group. In clinical practice, less than half of patients identified a stroke onset, probably due to difficulty relating the depictions of stroke symptoms to the real situation (Teuschl & Brainin, 2010; Yin et al., 2016). In addition, there was no difference in SRR and CAR among surgery group with or without stroke history during survey. This implied that the causes/results of surgery procedure did not affect individuals' response to stroke. Therefore, we propose that stroke education, including stroke recognition and calling an ambulance, be a part of routine care for patients receiving cardiovascular interventions.

In real‐world survey of acute stroke patients arriving at hospital, the utilization rate of EMS in rural area was markedly lower than that in urban China (9.9% vs. 18.3%–23.1%; Jin et al., 2012; Yin et al., 2016). Compared with urban area, there were higher inefficiency of medical service system and poor allocation of healthcare resources in rural China (Chen, Yin, & Xie, 2014; Zhai et al., 2017; Zheng, Gong, & Zhang, 2019). And inequities of emergency medical services also existed across regions (Yan et al., 2017). The system‐level problem of China—poor access to medical system—might decrease rural individual's intent to calling EMS, even in those with cardiovascular surgery experience.

Studies have demonstrated poor control of cardiovascular risk factors and low adherence to stroke secondary prevention efforts in China (Gao et al., 2013; Lu et al., 2017; Pan et al., 2016; Peng et al., 2014; Qin et al., 2016). Even for patients with a history of cardiovascular surgeries, therapy compliance was still unimproved (Halle et al., 2018).We assume that they may be unaware of, and underestimate the importance of, controlling risk factors to reduce stroke incidence (O'Donnell et al., 2016; Soroush et al., 2017). Considering the high prevalence of underlying diseases, it is necessary for cardiovascular surgeons to educate patients that surgery alone does not remove risk of vascular events and that continuous and good control of risk factors is vital to decrease postoperative stroke risk (Hobeanu et al., 2017; Kumar et al., 2017; O'Donnell et al., 2016).

There were several limitations in our study. First, the surgery population only involved those with previous cardiovascular surgery, and other surgeries which might influence patients' behaviors were unknown. In addition, the effect of surgery on calling EMS might be biased by the limited number and proportion of the surgery group. We assume that the surgery group would probably show significantly higher odds of calling EMS at the time of stroke onset, if more individuals with cardiovascular surgery experience were included in our multivariable analysis. Therefore, it is too simplistic to say that the odds of calling EMS were similar between those with and those without a cardiovascular surgical history. Second, the interval between surgeries and answering the questionnaires was unclear; thus, the impact of surgery may be biased by the heterogeneity of the interval. We concluded that patients with a recent surgical history may prefer to call an ambulance. Finally, the rates of stroke recognition, calling an ambulance, and their associated factors could be biased by the multistage nonrandom sampling design and selection from CNSSS (Li et al., 2019). This survey was also limited to recall bias, as participants might misremember the surgery information in the long ago.

In summary, different from the conventional perceptions, despite having markedly higher prevalence of cardiovascular risk factors, patients with a history of cardiovascular surgery showed similar ability to identify stroke onset and were not significantly better at calling an ambulance, compared with those without such history. Therefore, enhanced education programs on recognizing and responding to stroke onset should be provided to patients receiving cardiovascular surgery, as well postoperative education on the importance and benefit of good control of risk factors.

## CONFLICT OF INTEREST

Craig Anderson is employed by The George Institute China and has a National Health and Medical Research Council (NHMRC) of Australia grant. He is also a consultant for Takeda China and Amgen. The other authors declared no potential conflicts of interest with respect to the research, authorship, and/or publication of this article.

## AUTHOR CONTRIBUTION

B P., LD W., and LY C. designed the study. SD L., CD Y., GL S., C A., and B P. analyzed the data. CP G. finished data collection and management. SD L. wrote the paper. LY C., B P., and C A. revised the paper.

## Data Availability

Research data are not shared.
