# Supplementary material for: Cardiovascular surgery experience does not significantly improve patients' response to stroke
Source: Brain Behav. 2019 Sep 12;9(10):e01405. doi: 10.1002/brb3.1405 (PMC6790311; doi:10.1002/brb3.1405)
Supplement: Supplementary file 1 [file BRB3-9-e01405-s001.docx]

**SUPPLEMENTAL MATERIAL**

1. **List of the FAST-RIGHT Investigators and Coordinators**

Bin Peng, MD (PI), Shengde Li, MD, Li-Ying Cui, MD, Nan Jiang, MD, Yuehui Hong, MD, Peking Union Medical College Hospital, Department of Neurology, Beijing; Longde Wang, MD, Stroke Control Project Committee, The National Health Commission, Beijing; Craig Anderson, MD, PhD, Neurological and Mental Health Division, The George Institute for Global Health, Faculty of Medicine, University of New South Wales, Sydney, Australia; The George Institute for Global Health, Peking University Health Science Center, Beijing, China; Chengdong Yu, MD, Guangliang Shan, MD, Chinese Academy of Medical Sciences, Institute of Basic Medical Sciences, Department of Epidemiology and Statistics, Beijing; Weidong Liu, MD, Liaocheng People’s Hospital, Neurosurgical Department, Liaocheng, Shandong; Jian Li, MD, Affliated Hospital of Weifang Medical University, Neurology Department, Weifang, Shandong; Chunpeng Gao, MD, Dalian Municipal Central Hospital, Disease Control and Prevention Offce, Dalian, Liaoning; Suiqiang Zhu, MD, PhD, Huazhong University of Science and Technology, Tongji Hospital of Tongji Medical College, Department of Neurology, Wuhan, Hubei; Ping Xu, MD, Changde First People’s Hospital, Department of Neurology, Changde, Hunan; Tiemin Wei, MD, the Central Hospital of Lishui, Department of Cardiology, Lishui, Zhejiang; Yun Luo, MD, First People’s Hospital of Jiujiang, Department of Cardiovascular, Jiujiang, Jiangxi; Shengli Chen, MD, Chongqing Three Gorges Central Hospital, Department of Neurology, Chongqing; Dan Liu, MD, Jingmen First People’s Hospital, Jingmen, Hubei; Dongmei Xie, MD, Liuzhou Worker’s Hospital, Liuzhou, Guangxi; Dong Xu, Ningxia People’s Hospital, Yinchuan, Ningxia; Fei Wei, MD, Yichang Center People’s Hospital, Yichang, Hubei; Guanghui Wu, MD, Ningde City Hospital, Ningde, Fujian; Hongyan Li, MD, The People’s Hospital of Xinjiang Uygur Autonomous Region, Urumqi, Xinjiang; Hua Luo, MD, Affliated Hospital of Southwest Medical University, Luzhou, Sichuan; Jie Min, MD, The First People’s Hospital of Jingzhou, Jingzhou, Hubei; Jinhai Tang, MD, Jiangsu Province Hospital, Nanjing, Jiangsu; Jun Sun, MD, Wenzhou Central Hospital, Wenzhou, Zhejiang; Luoqing Li, MD, The First People Hospital of Yueyang, Yueyang, Hunan; Qi Yao, MD, Xinyu People’s Hospital, Xinyu, Jiangxi; Shilin Liu, MD, Pingxiang People’s Hospital, Pingxiang, Jiangxi; Wei Shi, MD, Affliated Hospital of Nantong University, Nantong, Jiangsu; Wei Yan, MD, The First People’s Hospital of Kashgar, Kashgar, Xinjiang; Xiaofei Yu, MD, Shuguang Hospital Affliated to Shanghai University of Traditional Chinese Medicine, Shanghai; Xiaopeng Luo, MD, Zhuzhou Central Hospital, Zhuzhou, Hunan; Xiaoxiang Peng, MD, Third people’s Hospital of Hubei Province, Wuhan, Hubei; Ya Zhang, MD, Dali Bai Autonomous Prefecture People’s Hospital, Dali, Yunnan; Yang Gao, MD, Yancheng City First People’s Hospital, Yancheng, Jiangsu; Ye Peng, MD, Harrison International Peace Hostipal, Hengshui, Hebei; Yongling Xue, MD, Qujing First People’s Hospital, Qujing, Yunnan; Zhi Lin, MD, Central People’s Hospital Of ZhanJiang, Zhanjiang, Guangdong; on behalf of the FAST-RIGHT study group.

1. The structure of FAST-RIGHT questionnaire with additional 4 questions about stroke awareness (Li et al., 2019)
2. **FAST-RIGHT**
3. Which disease do the following symptoms probably mean? (Facial droop, Arm weakness, Speech disturbance)

- Correct (Participant should give an answer like “stroke”, “cerebral infarct”, “Cerebral thrombosis”, “ cerebral hemorrhage”, “ cerebral embolism”, “ apoplexy”)
- Incorrect（none of above）

1. What will you do when encountering above-mentioned symptoms? (Single selection)

- Self-observation at home (incorrect)
- Call the family, wait for them, then go to hospital (incorrect)
- Call emergency phone immediately (correct)

1. Have your relatives or colleagues suffered from these diseases?:Yes/No
2. How do you get such information? (Single selection/multiple Choice)

Newspaper/TV/Broadcast/Wechat/Internet/popular science and technology (PST) activities

1. **The other relative factors in this survey: brief introduction**

- Basic Information
- Demographic information (age, sex, education, family member, marriage, career, living status, annual income, medical insurance)
- Address (province)
- The status of conducting questionnaire (by self /others/phone)
- Lifestyle
- Smoking (Yes/No/Quit/Years)
- Drinking (Yes/No/Quit/Years)
- Exercise habit (often/less frequency)
- Dietary habits
- Family history (stroke, coronary artery disease [CHD], hypertension, diabetes mellitus, dyslipidemia]
- Diseases and management during survey (2015-2017)
- cerebrovascular disease [new stroke, types, Outpatient/Inpatient, mRS ]
- Coronary Heart Disease [new CHD, atrial fibrillation [AF]/drugs]
- Hypertension [drug adherence, monitoring frequency, qualification rate]
- Diabetes Mellitus [drug adherence, monitoring frequency, qualification rate]
- Dyslipidemia [drug adherence, monitoring frequency, qualification rate]
- Physical examination (body mass index [BMI], blood pressure [BP], cardiac auscultation)
- Grade of stroke risk

Hypertension Diabetes mellitus Dyslipidemia AF

BMI-Obesity Smoking Lack of exercise

Family history History of stroke History of transient ischemic attack (TIA)

Five levels: Stroke TIA High-Risk Moderate-Risk Low-Risk

- EEG, serum test (glucose, lipid, homocysteine), carotid artery ultrasound for new and prior high-risk, stroke and TIA group.
- Surgery and interventional therapy during this survey
- Carotid artery [CAS, CEA, Extracranial-Intracranial Bypass]
- Coronary artery [PCI, CABG]
- Surgery/Interventional therapy for Intracranial hemorrhage [Yes/No]

1. Definition of Risk Factors for Stroke(Li et al., 2019)

| **Risk factor** | **Criteria** |
| --- | --- |
| Smoking | ≥6 months in life (accumulative or consecutive) |
| Alcohol drinking | ≥100mL spirit alcohol >3 times per week (self report) |
| Hypertension | 1. Systolic BP ≥140mmHg or diastolic BP ≥90mmHg, or taking BP-lowering drugs   OR   1. ABPM: 24 h mean BP ≥130/80 mmHg, or daytime BP ≥135/85mmHg, or nocturnal BP ≥120/70mmHg   OR  3. Home BP monitoring ≥135/85 mmHg(self report) |
| Dyslipidemia | LDL-C ≥1.8mmol/L, or HDL-C <1.04mmol/L, or TC ≥6.22mmol/L, or TG≥2.26mmol/L |
| Diabetes | 1. Fasting glucose ≥7.0 mmol/L or non-fasting blood glucose ≥11.0mmol/L with diabetes mellitus symptoms   OR  2. Fasting glucose ≥7.0mmol/L or non-fasting blood glucose ≥11.0mmol/L more than two times without typical DM symptoms  OR  3 .OGTT (75g glucose): 2 h blood glucose ≥11.0 mmol/L  OR   1. Taking glucose-lowering drugs |
| Significant overweight or obese | BMI ≥26.0 kg/m^2^ |
| AF | Either a history of persistent AF or supported by past ECG or ECG examination in this survey and confirmed by a cardiologist |
| Physical exercise | 1. ≥ 30 min of medium strength and above exercise every time, >3 times per week   OR  2. Moderate or heavy manual workers |
| Family history of stroke | Any parent or sibling with stroke; further inquiry and confirmation by a neurologist |
| History of stroke. | Either neurological deficit symptom at onset or symptomatic lacunar cerebral infarction on imaging, confirmed by a neurologist |
| TIA | Sudden onset focal/global neurological deficit lasting less than 24h, usually alleviating within 30minutes, excluding non-angiogenesis, and confirmed by a neurologist |

1. Calculation of level of stroke risk(Li et al., 2019)

| **Level** | **Definition** |
| --- | --- |
| Stroke | Stroke in previous or this survey |
| TIA | TIA in previous or this survey, except stroke |
| High-risk | ≥3 stroke risk factors, except stroke or TIA |
| Moderate-risk | 1. ≤2 stroke risk factors and with 1 being hypertension, AF/ valvular heart disease, or diabetes.   AND  2. without stroke and TIA |
| Low-risk | 1. ≤2 stroke risk factors and without 1 of hypertension, AF / valvular heart disease, or diabetes.  AND  2. without stroke and TIA |
| Risk factors | Hypertension, dyslipidemia, diabetes, AF, valvular heart disease, significantly overweight or obese, lack of exercise, family history of stroke, smoking |

Table S1. Missing data for each variable about stroke survivors

| Subgroup | Surgery | Non-surgery |
| --- | --- | --- |
| Age^†^ | 0 | 41 |
| Sex | 0 | 0 |
| Site | 0 | 0 |
| Region | 0 | 0 |
| BMI^‡^ | 0 | 144 |
| Education | 0 | 5 |
| Personal annual income | 1 | 49 |
| Living status | 1 | 49 |
| Children number | 1 | 238 |
| Stroke amongst people around them | 0 | 2 |
| Number of avenues taken to participate | 0 | 31 |
| Smoking status | 0 | 1 |
| Level of exercise | 0 | 1 |
| Family history of stroke | 0 | 70 |
| History of cerebral vascular disease | 0 | 10 |
| History of heart disease | 0 | 4 |
| History of hypertension | 0 | 5 |
| History of diabetes | 0 | 7 |
| History of dyslipidemia | 0 | 5 |
| Level of stroke risk^§^ | 0 | 16 |

^†^ Age ≥100 was classified as missing

^‡^ BMI >50 or <10 was classified as missing

^§^BMI >50 or <10 was not classified as missing data to calculate level of stroke risk.

**Table S2. Stroke recognition rate (SRR) and correct action rate (CAR) in urban and rural areas stratified in subgroups of non-surgery and surgery**

|  | SRR  n/N (%) | P value | CAR  n/N (%) | P value |
| --- | --- | --- | --- | --- |
| Non-surgery  Site |  | <0.0001 |  | <0.0001 |
| Urban | 77491/89868 (86.2) |  | 63071/89868 (70.2) |  |
| Rural | 74549/96113 (77.6) |  | 49891/96113 (51.9) |  |
| Surgery  Site |  | 0.3292 |  | 0.0353 |
| Urban | 100/115 (87.0) |  | 92/115 (80.0) |  |
| Rural | 58/71 (81.7) |  | 47/71 (66.2) |  |

N: total in every variable; n: number for recognizing stroke/correct action to stroke.

Non-surgery: Individuals without cardiovascular surgery

Surgery: Individuals with cardiovascular surgery

Figure S1. Data preparation and cleaning process

**Non-surgery group**: 185981

^†^ Virtual population to test the data management system.

^‡^ To ensure the accuracy, integrity, and creditability, residents finishing questionnaires via phone in CNSSS was not included in FAST-RIGHT study.

Delete: Missing data: 1556

**Surgery group:** 186

Delete: 2437

**Raw data in primary survey (N=243 279)**

Delete: 51126

**Data according to age standard (N=240 842)**

**Data of finishing face-to-face interview by oneself (N=189 716)**

**Final dataset (N=187 723)**

Delete: 1993

Step7: Did not answer question of stroke recognition (n= 1991)

Step8: Did not answer question of response to stroke (n= 2)

Step1: Death (n= 2413)

Step2: Test population^†^ (n=5)

Step3: Age < 40 years (n=13) and age ≥ 146 year (n=6)

Step4: Loss of follow-up (n= 6338)

Step5: Finish interview by others (n= 7240)

Step6: Finish interview via phone^‡^ (n= 37548)

**Figure S2. Distribution of stroke risk level between sugery and non-surgery groups**


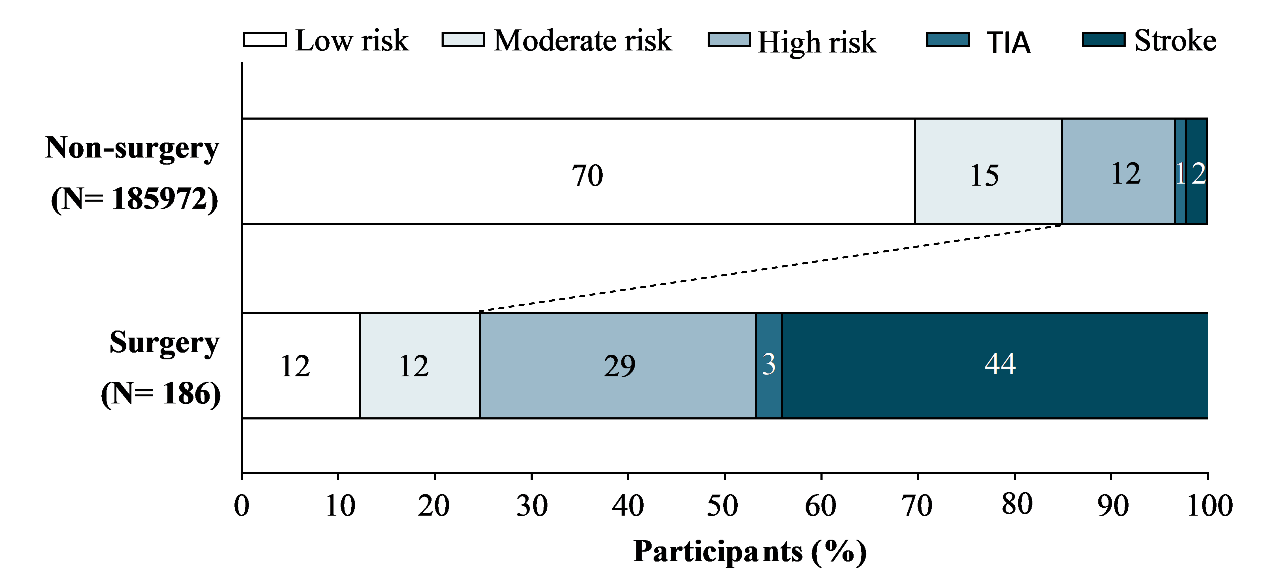


Stroke includes ischemic stroke, cerebral hemorrhage, and subarachnoid hemorrhage.

**Reference**

Li, S. D., Cui, L. Y., Anderson, C., Zhu, S. Q., Xu, P., Wei, T. M., . . . Coordinat, F.-R. I. (2019). Public Awareness of Stroke and the Appropriate Responses in China A Cross-Sectional Community-Based Study (FAST-RIGHT). *Stroke, 50*(2), 455-462. doi:10.1161/Strokeaha.118.023317
